# Supplementary material for: Are carbon emissions trading and green financial instruments synergistic? -Comprehensive quantitative research based on content analysis
Source: PLoS One. 2024 Mar 7;19(3):e0298601. doi: 10.1371/journal.pone.0298601 (PMC10919601; doi:10.1371/journal.pone.0298601)
Supplement: S1 Data — (ZIP) [file pone.0298601.s001.zip › Data/地级市Z_F工作报告.zip!/┴╔─■/╔≥╤⌠╩╨/└┤╫╘╣1⁄2╓┌║┼í╛┬φ┐╦╩2╛▌═°í┐_2007.docx]

各位代表，同志们：

　　现在，我代表市人民政府向大会作工作报告，请予审议，并请各位政协委员提出意见和建议。

　　一、2006年政府工作回顾

　　2006年，在省委、省政府和市委的正确领导下，在市人大的依法监督和市政协的民主监督下，市政府带领全市人民，继续坚持“工业立市”方针，紧紧围绕“两个不低于”和“三大目标”，开拓创新，奋力拼搏，圆满完成了市十三届人大四次会议确定的各项任务，实现了我市“十一五”时期的良好开局。

　　——预计全市地区生产总值实现2482.5亿元，增长16.5%，创14年来最高增幅；

　　——全社会固定资产投资完成1790.3亿元，再攀新高，增长31.3%；

　　——社会消费品零售总额实现1048.7亿元，增长14.6%，为5年来最高增速；

　　——实际利用外资实现30.4亿美元，增长43.4%，首次突破30亿美元；

　　——非公有制经济实现增加值1539亿元，占全市地区生产总值62%，已经成为拉动全市经济增长的主导力量；

　　——地方财政收入完成 236.4亿元，增长30.3%，连续6年保持30%以上速度增长；

　　——城市居民人均可支配收入达到11651.4元，增长15.4%，是11年来增长最快的一年；

　　——农民人均纯收入达到5712元，增长13.1%，连续保持较快增长。

　　主要经济指标增幅在全国副省级城市中继续名列前茅。

　　过去的一年，是全市经济持续快速增长，社会全面协调发展的一年；是招商引资取得显著成果，对外影响不断扩大的一年；是城市建设步伐进一步加快，综合实力和服务功能迅速提高的一年；是群众生活水平明显改善，和谐沈阳建设取得重大进展的一年；也是全市人民努力拼搏，对未来更充满希望和信心的一年！

　　（一）工业快速健康发展，整体实力和水平不断提升

　　去年，全市工业继续保持强劲发展势头。规模以上工业实现增加值929.3亿元，增长32.5%，在全国副省级城市中跃居第7位。经济效益大幅提高，实现利税178亿元，增长51%，利润71亿元，增长65%；经济效益综合指数160%，提高11.4个百分点，历史性地扭转了长期以来我市工业利润与利税增幅低于产值增幅的局面。

　　工业固定资产投资和新开工项目创历史新高。工业固定资产投资实现694.3亿元，增长36.1%。新开工投资500万元以上工业项目1370个，化工集团50万吨CPP等20个投资10亿元以上的重大项目陆续开工，为工业持续快速增长提供了有力支撑。

　　先进装备制造业基地建设取得新突破。装备制造业完成产值1647亿元，增长46.7%。汽车产量达到26.7万辆，增长92.7%。华晨金杯、特变电工等骨干企业产值增幅均达50%以上。铁西区装备制造业核心地位进一步突出。机床、沈鼓等重点企业完成搬迁改造，形成了新的生产能力。企业自主创新能力不断提高，开发了96项具有自主知识产权的重大装备产品。以盾构机等产品为主的地铁装备产业化步伐加快，填补了我市装备制造业发展空白。

　　工业发展空间不断拓展。全面启动了356平方公里细河经济区的开发建设，橡胶工业园等一批化工和冶金项目入驻。合署办公后的沈北新区发展势头强劲，玉米工业园等一批重大项目落地。欧盟经济开发区、满融开发区、航空工业园等特色产业园区建设全面展开。承载未来工业发展的“东汽、西重、南高、北农”格局基本形成。

　　国有企业改革取得重大进展。24户重点国有企业和227户国有中小企业转制任务基本完成。国有企业不良债权和股权回购等难点问题取得突破。

　　实践证明，我们坚持“工业立市”，一手抓项目开发、一手抓空间拓展的发展思路是完全正确的，巨大的成效日益凸显，进一步增强了全市人民加快工业全面振兴的信心。

　　（二）“开放年”活动成果丰硕，对外开放再上新台阶

　　世界园艺博览会盛况空前，累计接待游客1268万人次，总收入达4亿元。世园会的成功举办，增强了城市的凝聚力，极大地提高了沈阳的知名度和影响力。在此过程中展现出的“沈阳精神”，已经成为推动全市加快发展的强大动力和宝贵财富。

　　利用外资强劲增长，连续两年位居全省第一。实际调资额超1000万美元以上项目40个。对内招商成效显著，引进投资500万元以上项目1805个，调入资金536亿元。

　　进出口贸易稳步增长，实现进出口总额49.5亿美元，其中出口26.5亿美元，增长20%。企业“走出去”取得新进展，14个境外投资项目得到商务部批准。沈阳出口加工区正式封关运作，成为我市开拓国际市场的重要平台。

　　对外交往日益活跃。去年，继世园会、“制博会”和“韩国周”之后，全市又成功举办了第十四届世界生产力大会、中国风险投资论坛等重大国际活动，众多国内外嘉宾莅临沈阳。包括利比里亚总统、芬兰总理在内的重要外宾万余人相继来访。全年新开7条国际航线，空港旅客吞吐量达到530万人次。开放的沈阳正在走向世界。

　　（三）县域经济快速发展，新农村建设实现良好开局

　　农业生产在遭遇严重干旱的情况下，仍获得全面丰收。粮食总产量达到319.5万吨，肉、蛋、奶产量分别达到73.5万吨、44.8万吨、22万吨。

　　县域经济发展迅速。规模以上工业实现增加值346.7亿元，增长40.1%。新上产业集群项目605个，康平电厂等一批重大项目开工建设。规模以上农产品加工企业达2000余家，34万农户参与产业化经营，占农户总数的42%，初步形成了以粮、菜、畜、禽和林产品加工为主的产业体系。

　　农业产业结构调整步伐加快。优质、高效、特色经济作物面积增长20%，占农作物总面积的36%。新建富民经济小区534个，“一村一业、一乡一业”迅速发展，为经济区建设创造了条件。

　　加大了农村扶贫工作力度，筹集资金1亿元，新上扶贫开发项目275个。新增农村劳动力转移和输出14.2万人。

　　去年，是我市农村公路建设规模最大的一年。共投入6.1亿元，修建了县、乡、村三级公路1012公里。推广沼气等新型能源，开展“绿色村庄”活动，进一步改善了农村环境。

　　（四）第三产业日益繁荣，中心城市功能进一步拓展

　　第三产业持续快速发展，实现增加值1209.6亿元，增长13.1%。

　　城乡市场购销两旺，社会消费品零售总额首次突破千亿元。大型商业设施和城乡便民服务设施不断完善，方便了人民群众生活。

　　“金廊”项目建设稳步推进，新引进项目19个，卓展购物中心等10家国内外知名企业入驻，都市中央走廊形象初步形成。

　　物流、金融、会展等服务业规模不断扩大。全市物流企业达3100家，引进了日本邮船、德国TDS等国际知名第三方物流企业。金融机构本外币存款余额4000亿元，贷款余额2600亿元，分别增长8.2%和10.4%。新加坡大华银行、日本北海道银行和香港东亚银行相继落户。全年举办各类会展活动146项，交易额908亿元。

　　成功举办了“国际旅游节”、“第二届中国清文化节”等大型节会活动。接待国内外游客4553万人次，增长30.1%，创历史新高。

　　房地产市场平稳健康发展。我市房地产市场投资、销售、房价等主要指标，在15个副省级城市综合评价中名列第一。

　　辽宁中部城市群经济区建设取得新进展。本溪-辽阳-辽中高速公路开工建设，区内高速公路网络日趋完善。劳动力、人才、产权交易和旅游等共同市场建设步伐加快。“制博会”、“韩国周”已成为七城市招商引资的共同平台。

　　（五）城市环境明显改善，建设和管理水平实现新飞跃

　　去年，城市建设和管理经受住了世园会等重大国际活动的检验，跃上一个新台阶。全年城建完成投资155亿元，增长26%。

　　以拓展“四大发展空间”为重点，加快了“沈西工业走廊”、沈北新区、棋盘山旅游风景区、大浑南地区规划建设步伐，提高了城市未来发展的承载能力。

　　沈西、沈北开发大道和胜利南大街等主要道路全面建成通车，建设了兴华公铁地道桥等10座桥梁，沈康高速公路等重大项目启动，进一步完善了全市交通网络体系。地铁1、2号线和奥体中心等重大基础设施工程进展顺利。

　　实施大气环境综合整治，城市空气质量优良天数达320天。对浑河、南北运河环城水系进行治理，完成了细河源头24公里城市段清淤工程。建设完善了西部、北部和仙女河污水处理厂，全市污水处理率达到73%。

　　以办好世园会为契机，对重点广场、街道进行了高水平绿化、美化、亮化，城市面貌发生了巨大变化。深入开展“知荣明耻，文明出行”活动，市民文明素质明显提高。

　　（六）科技创新取得突破进展，创新型城市建设全面启动

　　以去年全市科技工作会议为标志，我市经济工作的着力点开始由结构调整向自主创新转变。

　　科技创新“六大突破”取得显著进展。全年实现高新技术产值1500亿元，增长36%；取得科技成果3185项，增长36%。镗铣加工中心等6种产品荣获“中国名牌”称号，一年新创名牌数量超过历年累计总和。

　　浑南新区高新技术产业发展迈出坚实步伐。加快了高新技术产业带规划建设，进一步拓展了高新技术产业发展空间。全市新增高新技术企业329家、科技型企业1135家，孵化面积达到90万平方米，成立投融资机构9家，引进海外高层次人才2500人次，有力地促进了全市的科技创新。

　　企业自主创新主体地位进一步确立，研发能力不断增强。规模以上企业共投入研发资金23亿元，是历年投入最大的一年。航发科技、远大铝业等重点骨干企业加快了产学研合作步伐。东软集团的软件和数字医疗技术达到国际先进水平。高档数控机床等13个重大科技专项进入实施阶段。搭建了高水平的动漫产业等技术服务平台，启动了东北亚软件基地、东北亚农业创新成果转化基地等对外合作基地建设。科技创新为推动沈阳振兴提供了新的动力。

　　（七）社会事业全面发展，民生工程扎实推进

　　全市在科学发展观指导下，坚持以人为本，着力推进各项社会事业全面协调发展。

　　在全国率先实施义务教育均衡发展，对全市106所中小学进行综合改革，加大教师交流力度，促进了教育公平。新建农村九年一贯制学校20所，在农村中小学实施远程教育，覆盖率达100%。6所万人职业学校建设进展顺利，市装备制造工程学校已投入使用。

　　文化事业更加繁荣。新建和改扩建364个基层文化活动场所，以广场文化为主的群众文化生活丰富多彩。成功举办了第六届沈阳艺术节。沈阳京剧院进入全国重点京剧院团行列，沈阳金融博物馆建成开馆，提高了全市文化事业发展水平。

　　群众体育蓬勃开展，竞技体育水平不断提升。投入3000万元为1124个社区安装了健身器材。国内一流、可容纳万人的沈水湾健身乐园投入使用。我市运动队在省十运会创造了金牌总数、奖牌总数、团体总分三项第一的纪录。

　　去年，是我市出台惠民政策最多、财政投入最大、群众得到实惠最多的一年。市财政投入43.8亿元，重点用于就业、社保、低保和新型农村合作医疗等方面，帮扶困难群体提高生活质量和水平。

　　加大了就业安置工作力度。新增就业15万人，零就业家庭全部实现至少一人稳定就业，城镇登记失业率控制在5.1%。

　　社保扩面工作取得新进展，养老、医疗、失业、工伤、生育保险参保人数均大幅增长。将被征地农民纳入社会保障体系。建立了农民工工资保障金制度。

　　加大了社会救助投入，较大幅度地提高了城市最低工资、失业保险、城乡低保和五保供养标准，70万企业退休职工平均养老金每月提高了155元。对城乡困难群体实施“两节”期间临时救助。对全市31万名义务教育阶段的农村学生和城镇贫困家庭学生实行了“两免一补”。

　　公共卫生体系建设进一步加强。新型农村合作医疗参合农民增至210万人，参合率提高到91.6%。社区卫生服务网络不断完善，全面实施城市贫困人口医疗救助，有效缓解了“看病难、看病贵”问题。

　　对6.1万户棚户区住宅进行了拆迁改造。市政府确定的为城乡群众办好24件实事任务全面完成。

　　（八）坚持依法行政，民主法制建设不断加强

　　全市各级政府认真接受人大依法监督和政协民主监督。市政府坚持按季度向市人大和市政协报告、通报工作。全年办理人大代表建议1061件，政协委员提案796件，办复率达100%。

　　市政府向市人大提报了《沈阳市安全生产条例》等8件立法草案，出台了《沈阳市中小学用地保护规定》等16件政府规章。认真实施《行政监察法》，深入开展行政执法监察和效能监察。

　　各级信访部门认真做好群众来信来访接待处理工作。

　　治安形势进一步好转。严厉打击了境内外敌对势力和“法轮功”等邪教组织的渗透破坏活动，确保了国家安全和社会稳定。开展“打黑除恶”专项斗争，增强了市民的安全感。

　　此外，人口与计划生育、统计、审计、物价、参事、民族、宗教、广播、电视、新闻、出版、侨务、质量技术监督、食品药品监督、气象、民兵、预备役、双拥、防震减灾、人防、档案、修志、文史、残联、红十字会和老龄工作也都取得了新成绩。

　　各位代表，过去一年我市经济和社会事业发展取得的突出成就，是省委、省政府和市委正确领导的结果，凝聚着沈城740万人民的心血和汗水。在此，我代表市政府，向为沈阳发展作出无私奉献、付出辛勤劳动的全市各族人民致以亲切问候和崇高敬意！向给予政府工作积极支持与监督的人大代表、政协委员，各民主党派、工商联和无党派人士、各人民团体，向参与沈阳发展建设的中央和省在沈单位、驻沈部队指战员、武警官兵和在沈工作的外籍人士、港澳台同胞、海外侨胞及国际友人表示衷心感谢！

　　总结过去的工作，我们清醒地看到我市经济社会发展仍面临许多困难和问题，政府工作还存在一些明显不足和薄弱环节。一是抓第三产业发展力度不够，与建设区域中心城市目标要求还有较大差距。二是科技创新能力不强，科技成果产业化水平较低，经济增长方式有待于进一步转变。三是外贸依存度较低，参与国际市场竞争能力还需要进一步提高。四是政府职能有待于进一步转变，一些政府部门行政效能、办事效率不高，全市发展软环境仍需改善。对此，我们要高度重视，认真研究，切实加以解决。

　　二、2007年政府工作安排

　　2007年是我市全面实施“十一五”规划，实现“三大目标”的关键之年。在新的一年里，全市发展面临着诸多机遇与挑战。国内城市在新一轮经济增长中竞相发展，地区间竞争日趋激烈，不进则退、慢进亦退。

　　我们必须增强大局意识、机遇意识、忧患意识，把全市发展置于全国发展的大格局中去审视，放在省委、省政府对沈阳提出的发展要求上去谋划，站在城市功能新的发展定位上去思考。

　　要继续围绕实现“三大目标”和完成“两个不低于”任务，抓住东北振兴和沿海开放双重机遇，把项目建设和环境建设放在更加突出的地位，解放思想，开拓创新，以超常思维实现超常发展，使经济总量和综合竞争力跃上新台阶，力争在全省率先进入东部沿海发达地区行列，为全面完成“十一五”规划确定的各项任务奠定坚实基础。

　　今年政府工作指导思想是：深入贯彻党的十六届六中全会、省第十次党代会和市第十一次党代会精神，全面落实科学发展观，紧紧抓住战略发展机遇，以改革开放为动力，以“创新年”活动为主线，着力推进体制机制创新和产业优化升级，继续保持经济又好又快发展的强劲势头，促进社会事业全面协调发展，把发展成果更多惠及人民群众，努力构建和谐沈阳。

　　今年全市经济和社会发展的主要预期目标是：地区生产总值增长15%，财政收入增长20％，固定资产投资增长16％，社会消费品零售总额增长13％，实际利用外资增长16.7%，城市居民人均可支配收入增长13％，农民人均纯收入增长13％。

　　（一）以“创新年”活动为主线，加快创新型城市建设

　　全面落实市科技工作会议提出的建设创新型城市任务，以科技创新和环境创新为核心，带动社会全方位创新，为经济社会发展提供持续动力，提高城市综合竞争能力。

　　加快推进体制机制创新。以深化企业产权制度改革为重点，进一步推进国有经济战略重组，全面完成国有企业改革任务。加强国有资产监管，强化对企业经济规模、运行质量、技术创新等方面的考核。全面推进厂办集体企业改革。继续大力支持非公有制经济发展。加快科技、文化等社会事业及城市公用事业改革。进一步转变政府职能，提高办事效率，创新工作方式，努力降低投资商务综合成本，营造宜于创业和发展的市场经济体制环境。

　　围绕科技创新“六大突破”，加快创新体系建设。高新技术产值实现2100亿元，增长40%。浑南新区要发挥全市科技创新的龙头作用，重点抓好芯片、数字医疗等重点项目和产业，加快软件服务业、动漫产业发展，积极申办国家级动漫产业基地。加快高新技术产业带建设步伐，提高产业发展承载能力。加快推进与中科院等院所的产学研合作，建立重型装备等30个产业技术平台。要在培育和引进创新型企业群体上取得新突破，重点培育年销售额超30亿元的科技领航型企业20家，新增科技型企业3000家。引进国内外人才1万人次、技术团队200个。加强创新型人才的培养和使用。要把孵化体系建设放在更加突出的位置，加快标准厂房等创新设施建设，确保新增孵化面积110万平方米，在孵科技企业3000家，提高科技成果产业化水平。进一步拓宽融资渠道，引进国内外创业投资机构和信用担保机构20家，加大对科技型中小企业上市融资的支持力度。

　　全面启动工业“2111”创新工程，提升工业整体发展水平。以设施农业等六种现代农业为重点，加快推进农业创新步伐。提高流通领域的信息化及电子商务应用水平，加快改造传统服务业。

　　推动资源节约型、环境友好型城市建设。大力发展循环经济，推进企业节能降耗工作，全市万元GDP综合能耗1.09吨标准煤，下降4.5%。在全市范围内广泛实施地源热泵技术，应用面积要达到1500万平方米。全面推行保温苯板等建筑节能新技术、新材料。加快推进中水回用和煤层气开发利用。加强污染物排放总量控制，全市主要污染物排放总量削减3%。

　　（二）着力抓好重大项目建设，保持工业快速发展势头

　　继续大力推进工业经济快速发展。规模以上工业增加值增长25%。

　　集中力量抓好重大项目和产业集群建设，全年必保新开工建设投资500万元以上工业项目1500个，投资10亿元以上重大项目24个。各区、县（市）至少引进一个投资50亿元以上的重大项目。突出抓好一批产值过百亿的重大项目。汽车零部件等3个产业集群要实现百亿产出。

　　铁西区、浑南新区、沈北新区、细河经济区及欧盟经济开发区要在引进重大项目上取得新突破。沈阳经济技术开发区、浑南新区要加快发展速度，向全国先进开发区行列迈进；沈北新区要积极争创国家级农业高新技术区；细河经济区要加快建设步伐，早出形象。全面加快满融开发区、胡台新城开发区、航空工业园等产业园区建设。

　　继续加快先进装备制造业基地建设。着力引进装备制造业重大项目，加快推进北方重工等一批骨干企业的搬迁改造。支持机床集团等10家企业的国际并购，组建输变电等12个成套设备公司，培育高性能机器人等50个具有自主知识产权的重大产品。加快14大类重大专项产业化步伐，争取数控机床、IC装备项目列入国家重大科技专项计划，不断提升装备制造业水平。

　　（三）加速农业产业化、工业化进程，全面推进新农村建设

　　把切实解决“三农”问题作为实现我市全面振兴和建设和谐社会的重要任务，认真贯彻工业反哺农业、城市支持农村和多予少取放活的方针，促进城乡协调发展。新农村建设要以县域经济为载体，以经济发展和环境建设为重点，加快向全国“百强县”目标迈进。今年，农村地区生产总值增长20%，财政收入增长30%。

　　突出抓好经济区建设。以发展“一村一业、一乡一业乃至一县一业”为突破口，加快农业产业化步伐，在经济区建设上取得新的成效。全市专业村要达到400个，特色产业区达到33个，初步建成10个各具特色的经济区。

　　县域经济要有新突破。继续加快县域工业发展，工业增加值不低于上年增长水平。确保新上投资500万元以上农产品加工企业300家，新开工产业集群项目500个，加快推进重大项目建设。

　　进一步优化农业结构，加快发展现代农业。高效作物面积增加到400万亩，设施农业面积增加到135万亩。畜牧业占农业比重提高到55%。

　　千方百计促进农民持续增收。新建富民经济小区400个。建立150个农民科技研修基地，加强对农民实用技术培训。新增农村劳动力转移和输出8万人。继续加大农村扶贫工作力度，投入资金1亿元，帮助贫困村脱贫致富。采取有效措施提高少数民族村农民收入水平。

　　大力改善农村环境。深入开展“绿色村庄”活动，“五一”前累计完成植树3000万株。修路进村，形成覆盖全市村庄的道路网络。引入市场机制，搞好农村垃圾无害化处理试点。

　　（四）提升服务业整体水平，推进区域中心城市建设

　　今年，全市要把发展现代服务业放在更加突出的位置，加快建设东北区域商贸金融中心。

　　继续优化商业布局，进一步加大对太原街、中街、长江街、西塔和东城等中心商贸区和22条商业街改造建设力度，培育滑翔等十大区域商贸中心。培育一批大型品牌市场，加速业态提升。引进投资500万元以上商业项目400个。

　　加快现代物流业发展。全面推进物流分拨、采购中心以及物流地区总部建设，重点培育第三方物流龙头企业20个，引进国内外大型知名物流企业10个。

　　增强区域金融服务能力。加快金融商贸开发区建设，尽快完成申报国家级开发区工作。大力发展风险投资和融资租赁等新型金融业务。加大外资银行引进力度，年内引进3家外资银行和4家非银行金融机构。重点推进3户企业境外上市。

　　保持房地产业健康发展。进一步规范房地产市场开发，调整住宅供应结构，保持市场活力，加大房贷投放力度，确保全年住房按揭贷款累计投放80亿元。

　　加快文化、体育产业发展。深化文化体制改革，重点发展传媒、发行、演艺和创意等文化产业。推出精品剧目，开拓演出市场。逐步提高文化、体育产业在服务业中的比重。

　　进一步加快旅游业发展。发挥后世园会效应，加强旅游资源开发，推进旅游目的地城市建设。全年接待国内外游客比上年增加500万人次，旅游业总收入增长20%。精心筹划各种展会，加快会展业发展。

　　着力推进辽宁中部城市群经济区一体化进程，务必在道路交通、通讯、物流、金融服务等方面取得突破性进展。加快经济区城际轨道交通系统的规划建设，实施沈阳和营口港的铁路、公路、管路对接，连通沈阳与周边城市开发区道路，实现区内经济互动发展。依托沈阳保税物流园区、桃仙空港和营口港在沈设置的陆港，加快经济区物流基地一体化建设。积极争取国家和省有关部门支持，与其他城市共享024区号资源。加快市商业银行重组和跨区域经营。

　　（五）以迎奥运为契机，加快城市建设步伐

　　城市建设要以提高综合服务功能和城市品位为重点，高起点规划一批标志性的重大基础设施项目。全年城建投资185亿元。

　　加快推进以“三大中心”为主的一批功能性、标志性重大项目建设，奥体中心主体工程年内要全部完工，国际会展中心要加快建设步伐，艺术中心要尽早启动建设。加快“金廊”建设，年内规划展示馆等10个项目交付使用。

　　加快地铁1、2号线建设，完成机场路扩建工程，尽快启动苏桃公路建设。推进浑河三好桥、南阳湖大桥工程建设进度，开工建设五爱街过浑河隧道。启动“沈西工业走廊”出海通道及京沈、沈大、沈丹高速公路联络线建设，进一步完善“四大发展空间”交通基础设施。

　　加大浑河南北滩地10公里景观带和百里运河综合改造力度，实施浑河、细河、蒲河、丁香湖及南北运河环城水系建设和水质改善工程，全市水面积率达到6.88%，形成环境优美的城市滨水休闲空间。

　　全面完成浑河南岸38.4公里的堤内绿化，新增绿化面积12.14平方公里。做好原动物园地区等精品绿地建设。在机关、学校、社区等单位开展建设“绿色庭院”活动，新建100块城区街头绿地。

　　加强城市污水治理。实施污水处理建设工程和辉山明渠、七二四明渠整治工程。控制大气污染，大气优良天数保持在320天以上。

　　建立和完善城市管理长效机制，加大管理力度，提高管理水平，巩固市容环境整治成果。做好“国家环保模范城市”复检准备工作。

　　（六）加大招商引资力度，进一步扩大对外开放

　　坚持对外开放第一战略，把招商引资作为拉动全市经济持续快速发展的主要动力，继续保持沈阳对外开放强劲势头。实际利用外资实现35.5亿美元，外贸出口总额31亿美元。

　　加大招商力度，明确招商重点，创新招商方式。以引进世界500强和企业地区总部为重点，积极引进投资额1000万美元以上的重大项目。继续加大对内招商步伐，确保引进投资500万元以上内资项目1800个。

　　努力扩大外贸出口。大力引进出口加工型项目，做好向国家申报“装备制造出口创新基地”、“中国服务外包基地”工作，积极承接国外软件外包和服务外包业务，扩大服务贸易出口。

　　重点推进20户企业“走出去”，开展跨国经营。支持企业对外工程承包，参与国际合作与竞争。

　　做好“制博会”、“韩国周”等大型活动的组织创新，申请并办好“中国创新城市国际论坛”、“中国东北亚经济发展与合作会议”、“世界文化遗产与自然遗产博览会”等重大国际活动。

　　进一步做好对外友好往来工作，邀请更多的外国政要来访，争取更多的国家在沈设立领事馆。新开通5条国际航线，把沈阳建成东北地区航空枢纽港。

　　（七）全面推进社会事业发展，努力构建和谐沈阳

　　把促进社会发展和解决民生问题摆在更加突出的位置，继续加大投入力度，解决好人民群众最关心、最直接、最现实的利益问题，加快和谐社会建设。

　　全年新增就业15万人。进一步加强就业援助工作，重点帮扶困难群体就业，确保零就业家庭至少一人实现稳定就业。落实好“4050”灵活就业人员社保补贴政策。城镇登记失业率控制在5%以内。

　　进一步扩大养老、医疗、失业、工伤、生育等社会保险覆盖面，提高缴费率。加强社会救助体系建设，完善农村低保制度，做好城市低保家庭租房和采暖补贴发放工作。切实做好失地农民的就业和社会保障工作。建立稳定的社会劳动关系，进一步维护好农民工合法权益。支持慈善事业发展。

　　进一步完善城乡医疗服务体系。改造建设6所市属重点医院。加快社区卫生服务中心建设，达标率和覆盖率达到100%。完善新型农村合作医疗制度，覆盖率达到92%以上。加大政府投入力度，提高新型农村合作医疗筹资标准和农民医疗保障水平。加强乡镇卫生基础设施建设，健全农村三级医疗服务体系。

　　采取有力措施支持驻沈大学和科研院所发展。加快推进万人职业学校建设，市金融学校年内要投入使用。推进城乡义务教育均衡发展，加大对农村义务教育投入，新建20所九年一贯制学校。继续做好扶困助学工作，落实义务教育“两免一补”政策。

　　大力发展公益性文化事业，建立覆盖全社会公共文化服务体系。积极推进历史文化名城建设，做好历史文化遗址的修缮、保护工作。恢复建设二战盟军战俘集中营陈列馆等一批专题博物馆。

　　积极开展全民健身活动，进一步提高竞技体育水平。精心办好奥足测试赛，在全市开展迎奥运系列活动。

　　高度重视公共安全。完善应急管理体系，有效应对各种风险，做好重大疫情的监控和防范工作；完善食品监管体系，严格市场准入；完善社会治安防控体系，打击严重犯罪，降低发案率、提高破案率；完善安全生产监管体系，坚决遏制特大安全事故发生。

　　大力推进和谐社区、和谐村屯建设。不断健全新型社区管理和服务体制，完善社区公共服务。

　　今年继续为城乡群众办好20件实事。

　　（八）加强民主法制建设，努力建设服务型政府

　　认真实施《中华人民共和国各级人民代表大会常务委员会监督法》，健全向人大常委会报告工作机制，自觉接受人大法律监督和工作监督。主动接受政协的民主监督。认真办理人大代表的议案、建议和政协委员的提案。坚持依法行政，加强政府立法工作和行政执法监督。进一步畅通信访渠道，建立新的群众利益诉求、表达和维护机制。

　　加快政府职能转变，努力建设服务型政府，不断强化政府社会管理和公共服务职能。进一步深化行政审批制度改革，最大限度地下放管理权限，全面简化审批程序，不断提高审批效率。要切实转变和改进工作作风，杜绝以文件贯彻文件、以会议落实会议等现象，深入实际，调查研究，认真解决影响改革发展稳定的突出问题。坚决遏制文山会海和滥检查评比活动，进一步减轻基层和企业负担，从根本上改善全市发展软环境。

　　政府各部门要克服狭隘的部门利益和本位主义，摒弃陈旧的思维方式和工作方式，主动自觉地为全市发展大局服务。各级领导干部要切实加强作风建设，树立强烈的责任意识，敢于面对挑战，敢于承担责任，求真务实，不断创新工作。

　　加强公务员队伍建设，严格公务员管理，提高公务员行政能力。加强公务员勤政廉政教育，加大行政监察和对违纪违法案件查处力度，建设人民满意的公务员队伍。

　　各位代表，同志们，沈阳已经站在新的历史起点上。加快振兴，强市富民，是全市人民共同的心愿和期盼。在新的一年里，我们一定不负人民的重托和厚望，在省委、省政府和市委的正确领导下，励精图治，努力拼搏，乘势而上，以优异成绩迎接党的十七大胜利召开！
